# Importance of *Angomonas deanei* KAP4 for kDNA arrangement, cell division and maintenance of the host-bacterium relationship

**DOI:** 10.1038/s41598-021-88685-8

**Published:** 2021-04-28

**Authors:** Camila Silva Gonçalves, Carolina Moura Costa Catta-Preta, Bruno Repolês, Jeremy C. Mottram, Wanderley De Souza, Carlos Renato Machado, Maria Cristina M. Motta

**Affiliations:** 1 Laboratório de Ultraestrutura Celular Hertha Meyer, Instituto de Biofísica Carlos Chagas Filho, Universidade Federal do Rio de Janeiro, IBCCF, CCS, UFRJ, Cidade Universitária, Rio de Janeiro, RJ CEP 21941-590 Brazil; 2 Centro Nacional de Biologia Estrutural e Bioimagem, Rio de Janeiro, RJ, Brazil; 3 Department of Biology, York Biomedical Research Institute, University of York, Wentworth Way, Heslington, York YO10 5DD UK; 4 Laboratório de Genética Bioquímica, Departamento de Bioquímica e Imunologia, Instituto de Ciências Biológicas, Universidade Federal de Minas Gerais, Belo Horizonte, Brazil

**Keywords:** Parasite biology, Parasite genetics

## Abstract

*Angomonas deanei* coevolves in a mutualistic relationship with a symbiotic bacterium that divides in synchronicity with other host cell structures. Trypanosomatid mitochondrial DNA is contained in the kinetoplast and is composed of thousands of interlocked DNA circles (kDNA). The arrangement of kDNA is related to the presence of histone-like proteins, known as KAPs (kinetoplast-associated proteins), that neutralize the negatively charged kDNA, thereby affecting the activity of mitochondrial enzymes involved in replication, transcription and repair. In this study, CRISPR-Cas9 was used to delete both alleles of the *A. deanei KAP4* gene. Gene-deficient mutants exhibited high compaction of the kDNA network and displayed atypical phenotypes, such as the appearance of a filamentous symbionts, cells containing two nuclei and one kinetoplast, and division blocks. Treatment with cisplatin and UV showed that Δ*kap4* null mutants were not more sensitive to DNA damage and repair than wild-type cells. Notably, lesions caused by these genotoxic agents in the mitochondrial DNA could be repaired, suggesting that the kDNA in the kinetoplast of trypanosomatids has unique repair mechanisms. Taken together, our data indicate that although KAP4 is not an essential protein, it plays important roles in kDNA arrangement and replication, as well as in the maintenance of symbiosis.

## Introduction

The kinetoplast contains the mitochondrial DNA (kDNA) of trypanosomatids, which is arranged in a network of several thousand minicircles categorized into different classes and several dozen maxicircles that are virtually identical. Minicircles (0.5–10 kb) are physically connected to each other and also to maxicircles (20–40 kb) that are usually interwoven into the network periphery^1,2^. Maxicircle sequences encode components of the respiratory chain and ribosomal proteins, but first, posttranscriptional editing of the generated mRNA is required. This process is mediated in part by small noncoding guide RNAs (gRNAs) that are transcribed from minicircles^3,4^. The kDNA network is linked to the basal body through proteins that compose the tripartite attachment complex (TAC)^5^. Usually, loss of kDNA is associated with mitochondrial dysfunction, which makes this structure a potential chemotherapy target and diagnostic marker for trypanosomiasis^6–8^.

In contrast to most eukaryotes, mitochondrial DNA replication in trypanosomatids is regulated during the cell cycle, initiating immediately before nuclear DNA replication in S phase followed by network scission and kinetoplast division during the G2 phase. The duplication cycle of the kinetoplast occurs in four steps: kDNA synthesis; scission, when kDNA is cleaved into two networks; separation; and partitioning of kinetoplast between the daughter cells during cytokinesis^9^. The kDNA network replication is a complex and unusual mechanism that involves various enzymes, such as the mitochondrial topoisomerase II (mtTopo II), which detaches covalently closed minicircles from the network. Minicircle replication initiates at the kinetoflagellar zone (KFZ), which comprises the region between the kDNA facing the basal body and the inner mitochondrial membrane. At the KFZ, the minicircles duplicate as theta structures, by UMSBP, Pol 1B, and other proteins and subsequently migrate to the antipodal sites. At this kinetoplast region, a primase enables the synthesis initiation of new DNA fragments following kDNA replication that involves more than 100 enzymes, such as universal minicircle sequence-binding protein (UMSBP) and polymerases. Next, each newly replicated minicircle is reattached to the network by the mtTopoII, maintaining at least one nick/gap that is filled by proteins, such as Pol β-PAK and DNA ligase kα, prior to the network scission. Later, the duplicated network is separated by the basal body distance, since the kDNA is connected to it via the TAC structure. This minicircle replication model was primarily based on findings obtained with *Trypanosoma brucei* and *Crithidia fasciculata*^4^.

The kDNA arrangement varies according to species and developmental stages, ranging from densely packed fibers to a looser distribution in the kinetoplast matrix^10–12^. The proteins involved in this intriguing phenomenon have not been fully characterized. Kinetoplast-associated proteins (KAPs) are homologous to small basic histone H1-like proteins and nonhistone high-mobility group (HMG) box-containing proteins. KAPs have low molecular weights, are highly basic, are rich in alanine and lysine residues and contain a cleavable nine amino acid presequence involved in protein import to the kinetoplast in their amino-terminal region^13^. KAPs are involved in kDNA duplication, transcription, packing and topological remodeling^14–16^. KAPs can also bind to other proteins, such as UMSBP; in this case, they promote kDNA unpacking and facilitate the access of mtTopoII, which liberates minicircles from the network for replication^17^.

The first model used to study the roles played by KAPs was the monoxenic *Crithidia fasciculata*, where the disruption of the *KAP1* gene generated viable cells with a phenotype of highly condensed kDNA fibers, which was similar to that observed when trypanosomatids were treated with nalidixic acid, an inhibitor of prokaryote topoisomerase II^15,18^. When both *C. fasciculata* alleles for *KAP2* and *KAP3* were disrupted separately, no detectable phenotypes were generated, and the same lack of phenotypes was observed to heterozygous cells (*kap2⁄3*^+⁄−^), indicating a redundant function for these two encoded proteins. However, the double-knockout cells had notably slow proliferation, atypical cell morphology, an increased copy number of mRNAs encoding for ATPase and a significantly reduced respiration^15^. These first findings obtained with knockout cells indicated that KAPs were involved in distinct functions, such as kDNA arrangement and metabolism. Deletion of the *KAP3* gene was also performed in *Trypanosoma cruzi* by homologous recombination. Such null mutants did not exhibit changes in cell proliferation, differentiation, kDNA arrangement and infectivity, suggesting that this KAP is not essential for this parasite^19^. Later, the RNAi system was used to knockdown proteins associated with kDNA in *Trypanosoma brucei*. Downregulation of KAP6 promoted cell growth arrest and inhibition of covalently closed minicircle release, resulting in loss, shrinkage and disorganization of kDNA^20^.

Symbiont-harboring trypanosomatids (SHTs), such as *Angomonas deanei* (previously classified as *Crithidia deanei*^21^), coevolve in a mutualist relationship with a single bacterium that divides in synchronicity with other host cell structures and is usually observed close to the nucleus. During the protozoan cell cycle, the bacterium is the first DNA-containing structure to divide, followed by the kinetoplast and the nucleus^22–24^. The symbiont is a Gram-negative of the Alcaligenaceae family that contains a reduced genome, is enclosed by two membranes and has a very reduced peptidoglycan layer^25–27^. Such species has been used to study the kinetoplast which, in these cells, presents atypical shapes and a looser kDNA arrangement, which is more susceptible to topoisomerase inhibitors and DNA-binding drugs^11,18,28,29^. Recently, phylogenetic analysis showed that SHTs present an expanded repertoire of nuclear encoded KAPs and that genes for KAP4 and KAP7 are present in all trypanosomatid species analyzed to date^11^.

While mitochondrial DNA is subjected to the same damage sources as nuclear DNA, the reactive oxygen species (ROS) generated by the oxidative phosphorylation metabolism usually results in higher mutation rates in the mtDNA than does damage caused to nuclear DNA. In mammalian cells, base excision repair has been described as a restoration mechanism in the mitochondrion with the identification of several glycosylases, such as MYH, NEIL1, NEIL2 and UNG1, that are involved in the response of mtDNA to oxidative damage^30–33^. Other proteins, such as APE1, APE2, FEN1, and DNA2, were also detected, suggesting that all steps of this repair mechanism are present in the mitochondria of mammalians^34–38^. Mismatch removal activity was also identified in this organelle^39^, although it has not been determined which proteins are involved in this process and whether the same pathway is active in the nucleus. However, the most striking and unexpected feature in mammalian cells is the lack of DNA repair mechanisms to address UV- and cisplatin-induced lesions on the mtDNA^40–42^.

In trypanosomatids, some proteins involved in DNA repair have been described in both nuclear DNA and in kDNA metabolism. It was demonstrated that *T. cruzi* is able to remove oxidative lesions from both genomes, although damage to the kDNA remains higher than that in the nucleus^43–45^. This parasite contains DNA glycosylases that participate in the kDNA damage response^43,44^, as well as polymerases involved in the response to oxidative stress, such as Polβ, Polβ-PAK^46,47^ and Polκ, which are able to interact with intermediates of the homologous recombination^48^. Studies in *T. brucei* showed that the bloodstream form is able to deal with damage caused by cisplatin, hydrogen peroxide and methylmethanesulfonate (MMS), suggesting that DNA repair pathways are present in the parasite mitochondrion and that TbRad51 might be crucial to the response to alkylation lesions^49^.

In the present work, for the first time, we used the CRISPR-Cas9 system to analyze the role played by KAP in a trypanosomatid protozoan. The results demonstrated that *A. deanei* Δ*kap4* mutants have reduced proliferation and exhibit morphological and ultrastructural alterations. In KAP4 mutants, the kDNA network becomes highly packed and cells have atypical phenotypes including filamentous bacterium and atypical numbers of nuclei and kinetoplasts. Considering alterations in kDNA arrangement, gene deletion mutants were not more sensitive to cisplatin and UV treatment than wild-type protozoa, but these genotoxic agents interfered with cytokinesis in both cell types. Notably, cisplatin and UV lesions can be repaired in mitochondrial DNA, which suggests that there are unique DNA repair mechanisms in the trypanosomatid kinetoplast.

## Materials and methods

### Cell culture

The *Angomonas deanei* wild type (WT—ATCC 30255) strain was cultured in Warren´s medium^50^ supplemented with 10% fetal bovine serum. Protists were maintained by weekly passages by inoculating 10% of an established cell culture in fresh medium. WT and T7RNAPol-SpCas9 cell lines were grown at 28 °C for 24 h and cells with single or double deletions to *kap4* genes were grown for 48 h, both cases corresponded to the protozoan exponential growth phase. After this growth period, cells were used in assays or stored at 4 °C.

### Analysis of cell growth and viability

For the growth curve, the initial cell concentration was 1 × 10^6^ cells/mL, and counts were made every 24 h up to 72 h. Cell density was determined by counting live protozoa in a flow cytometer, where cell size was evaluated by detection of forward scatter on an SSA detector in a BD Accuri C6 flow cytometer (Becton Dickinson Bioscience BDB, San Jose, CA, USA). The relative growth rate (μ, expressed as h − 1) of the exponential phase was estimated by an exponential function $y=A{e}^{Bx}$, considering the parameters of culture cell density (cells/mL) vs culture time (h) of each strain, when B = μ. Such graphics only considered the cell density from 0 to 48 h of growth, which corresponds to the exponential phase, when all assays in this study were performed. Cell duplication time (DT) was calculated according to the formula $DT=\frac{ln2}{\mu }$.

To test cell viability, 5 × 10^6^ cells were washed once with filtered-sterilized PBS (phosphate-buffered saline) pH 7.2 and incubated for 10 min with 20 μg/mL propidium iodide (PI). After this step, 10,000 events per sample were collected, and the fluorescence was detected on an FL-2 filter (488/630). The percentages of viable and nonviable cells were determined using control assays of life and death, respectively. To check cell death, cells were fixed in 4% paraformaldehyde for 10 min, washed with PBS, pH 7.2 and subsequently incubated with propidium iodide (PI 1:100). To control for living cells, protozoa were washed in PBS, pH 7.2, but were not incubated with PI. Cell fluorescence was detected as previously described. In such viability assays, as well as in growth curves, cells were collected on a BD Accuri C6 flow cytometer (Becton Dickinson Bioscience BDB, San Jose, CA, USA) using the manufacturer software.

### Genotoxic treatment

WT and AdKAP4 mutants were compared by plating 1 × 10^7^ cells/mL in the presence or absence of genotoxic agents. For cisplatin treatment, cells were incubated with 150 and 300 μM of the inhibitor for 1 h, washed three times with PBS at pH7.2 and resuspended in fresh medium. UVC irradiation (254 nm) was performed with a germicidal lamp at a fluence rate of 1,500 µJ/cm^2^ (GS GeneLinker UV Chamber, Bio-Rad). For growth curves, in all conditions, the number of surviving cells was determined at 0 h (immediately before the treatment) and after 12 and 24 h of treatment, which corresponds to the *A. deanei* exponential phase^19^. Experiments were performed in triplicate. The cell number was determined in a hemocytometer chamber using the erythrosine vital stain (0.4% diluted in 1 × PBS) to differentiate living and dead cells. Only dead cells were stained, presenting a red color. The survival rate was calculated by comparing treated and control cells, which were employed as references (considered as 100%).

### Cell cycle analysis by flow cytometry

Protozoa were treated with cisplatin 150 and 300 μM for 1 h. Next, the cells were washed twice with PBS, pH 7.2, and the culture medium was replaced as described above. Protozoa were analyzed before treatment, as well as 1 h and 24 h after the incubation with the inhibitor. Approximately 5 × 10^6^ cells were pelleted, washed once with PBS and fixed in 0.25% paraformaldehyde at room temperature for 5 min. Next, the cells were permeabilized in 70% ethanol, in an ice bath, for 30 min and incubated with 100 μg/mL RNase and 25 μg/mL propidium iodide at 37 °C for 30 min. After this step, 10,000 events per sample were collected, and the fluorescence was detected on an FL-2 filter (488/630) on a BD Accuri C6 flow cytometer (Becton Dickinson Bioscience BDB, San Jose, CA, USA) using the manufacturer’s software. DNA histograms were analyzed with the same software.

### CRISPR-Cas9 gene editing

#### Protozoa transformation

*Angomonas deanei* transfections were performed by electroporation using the Amaxa 2B system program U-033 (Human T Cell Nucleofector™ Kit—Lonza), as previously described^24^. Cultures were immediately split into 2 populations, and recovered for 4 h at 26 °C before the addition of suitable antibiotics. Motile cells in both populations were counted and diluted for distribution in 96-well plates (200 µL of 1 or 0.5 cells/well). Clones were recovered after 5–8 days. *Angomonas deanei* T7RNAPol-SpCas9 was engineered using the pTB007 plasmid previously employed for *Leishmania* species, and SpCas9 expression was confirmed by Western blotting as in Beneke et al., 2017^51^. Transgenic lines were maintained in the following antibiotics and respective concentrations: G418 (250 µg/mL) and hygromycin (300 µg/mL).

#### CRISPR-Cas9 DNA fragment preparation

CRISPR-facilitated mutants were obtained by transfection of PCR fragments. The sgRNA sequence was obtained from EuPaGDT^52^, selected based on correct on-target sequence (ADEAN_000063100)^53^ and fewer *A. deanei* genome off-target hits, as well as sgRNA predicted activity. The sgRNA forward oligonucleotide is designed by flanking it with the T7RNAPol promoter (upstream) and the first 20 nucleotides of the SpCas9 scaffold (downstream). This oligo is combined with a universal primer containing the remaining sequence of SpCas9 backbone (OL00—Table 1). Amplification was performed in 20 µL using 0.2 mM dNTPs, 2 µM of each primer in Q5 reaction buffer and high-fidelity polymerase (NEB). The PCR program was set as 30 s at 98 °C followed by 35 cycles of 10 s at 98 °C, 30 s at 60 °C, and 15 s at 72 °C. The repair template fragments were produced using primers containing annealing sequences compatible with pPLOT and pT plasmids^51^ and 30 nucleotide homology arms at the 5′end of the oligonucleotide, both forward and reverse, for recombination upstream and downstream of the DNA double strand break (DSB), respectively, at the UTR of the gene. Fragments were amplified from 20 ng of pTNeo_v1^51^ using the same reaction buffer described above for sgRNA fragments in a final volume of 40 µL. PCR program was 10 min at 98 °C followed by 40 cycles of 30 s at 98 °C, 30 s at 60 °C, 2 min 15 s at 72 °C, and a final elongation step of 10 min at 72 °C. Products were run on 2% (sgRNAs) or 1% (repair templates) agarose gels in 0.5% Tris–Borate-EDTA (TBE) to confirm fragment amplification and expected sizes. Primer sequences are detailed in Table 1. DNA for transfection was prepared by combining sgRNA and repair templates followed by precipitation in a one-tenth volume of 3 M NaOAc, pH 5.5 and 2.5 volumes of ice-cold absolute ethanol, and washing in 70% ethanol thereafter. DNA was resuspended in 10 µL of molecular biology grade water and immediately transfected.

**Table 1:** List of oligonucleotides for CRISPR-Cas9 in *A. deanei*, including sgRNA, repair template and diagnostic PCR*. *Sequences are written in the 5′ to 3′ orientation.

Oligo name	Description	Sequence
OL00	Universal reverse primer for sgRNA amplification	aaaagcaccgactcggtgccactttttcaagttgataacggactagccttattttaacttgctatttctagctctaaaac
OL01	KAP4—5′ sgRNA primer	gaaattaatacgactcactataggCGGCGCTTACAGCATGTTTAgttttagagctagaaatagc
OL02	KAP4—3′ sgRNA primer	gaaattaatacgactcactataggTTTCTGCTGTTTCCACAGTTgttttagagctagaaatagc
OL03	KAP4—Upstream Forward primer	TACTCTTATTATAATTAGTTTTTTTATAAAgtataatgcagacctgctgc
OL04	KAP4—Downstream Reverse primer	TTTTTATTATTATTTGAATAGGTTTACCGCccaatttgagagacctgtgc
OL05	KAP4—Checking CDS presence/deletion/Neo integration (Forward)	GTCTCATAGGAAAAGTACAC
OL06	KAP4—Checking CDS presence/deletion (Reverse)	CGGCTTTTCTGCTGTTTC
OL07	Reverse at 5′ (ATG) of Neo resistance gene	ACTAGTATGGGATCGGCCATTGAACAAG
qPCRMitF	Forward qPCR primer for large mitochondrial fragment (approximately 10kB)	TTTTATTTGGGGGAGAACGGAGCG
qPCRMitR	Reverse qPCR primer for large mitochondrial fragment (approximately 10kB)	TTGAAA CTGCTTTCCCCAAACGCC
qPCRMitSmF	Forward qPCR primer for small mitochondrial fragment (250 bp)	CGCTCTGCCCCC ATAAAAAACCTT
qPCRNucF	Forward qPCR primer for large nuclear fragment	GAGGCACTGCATACCATTCAAG
qPCRNucR	Reverse qPCR primer for large and small nuclear fragment	GTGGTCCTTCTTTGTCAATTTCAC
qPCRNucSmF	Forward qPCR primer for small nuclear fragment	ATATACACGGGATAAAGGCCAGC

### Diagnostic PCRs

Genomic DNA (gDNA) was purified after clone cell culture amplification and kept under antibiotic selection, using the DNeasy Blood & Tissue Kit (Quiagen) following the manufacturer’s instructions. PCRs were set using 50 ng of gDNA using PCRBIO HS Taq Mix Red (PCR Biosystems) and 0.4 µM of primers to amplify the CDS locus or the integrated repair template containing the resistance marker gene (Neo). The oligonucleotides OL05 + OL6 were used to detect *KAP4* presence or absence, respectively. Oligonucleotides OL05 + OL07 were used to confirm integration of the repair template containing the neomycin (Neo) resistance marker at *KAP4* loci. The PCR program used was 5 min at 95 °C followed by 25 cycles of 30 s at 95 °C, 30 s at 55 °C, 20 s at 72 °C and a final elongation step of 5 min at 72 °C. Reactions were directly run in a 0.8% agarose gel in TBE to confirm genetic manipulation by comparing the presence or absence of WT and mutants PCR products. Primer sequences are detailed in Table 1.

### Fluorescence microscopy

#### DAPI staining

Protozoa were collected by centrifugation at 2000×*g*, washed once with PBS (phosphate buffered saline) pH 7.4, fixed in 4% paraformaldehyde in the same solution, and mounted on poly-l-lysine-coated circular microscope coverslips (14 mm diameter), next, the slides were washed with PBS and incubated with 10 μg/ml 4′,6-diamidino-2-phenylindole (DAPI, from Molecular Probes, Oregon, USA) for 10 min. After washing with PBS, slides were mounted using ProLong Gold (Molecular Probes), and visualized using a TCS SP5 confocal laser scanning microscope (Leica, Germany). Confocal images were obtained using an HCX PL APO 60 × objective for light microscope oil immersion with a numerical aperture of 1.4. Optical sections obtained from the whole cell were transformed into 2D images by maximum projection in the manufacturer’s software (LAS-X). The cellular patterns were determined by counting DNA-containing structures as nuclei, kinetoplasts and symbionts. Symbiont division was evaluated based on its form as described previously^22,24^. Analyses were based on counts of 1000 cells of WT and KAP4 mutants.

#### Immunofluorescence with anti-porin antibody

Protozoa were washed in PBS and fixed with freshly prepared 2% formaldehyde diluted in PBS, for 1 h. After fixation, cells were adhered to poly-l-lysine-coated microscope coverslips and permeabilized with 4% Nonidet P-40 (NP-40) diluted in PBS for 45 min. Slides were incubated in blocking solution containing 1.5% bovine serum albumin (BSA), 0.5% teleostean gelatin (Sigma Aldrich), and 0.02% Tween 20 diluted in PBS. Next, slides were incubated for 1 h with antibody produced against the symbiont porin^54^ diluted 1:10 in blocking solution. After that step, the cells were washed with PBS and incubated for 45 min with Alexa488-conjugated anti-mouse IgG (Molecular Probes, USA) diluted 1:200 in blocking solution. Slides were mounted using the anti-fading reagent ProLong Gold containing 5 μg/mL of DAPI (4′,6-diamidino-2-phenylindole, MolecularProbes). Serial image stacks (0.36-μm Z-increment) were collected at 64× (oil immersion 1.4 NA) on an Elyra PS.1 microscope (Carl Zeiss) and three-dimensional projections were obtained on the Zen Black program (Carl Zeiss).

#### In situ labeling of kDNA networks

Cells were centrifuged, washed, and fixed in 2% paraformaldehyde diluted in PBS for 5 min. Next, cells were adhered to poly-l-lysine-coated slides for 10 min and washed twice in PBS containing 0.1 M glycine for 5 min. After permeabilization in methanol for 1 h at 20 °C, cells were rehydrated with three washes in PBS for 5 min and incubated for 60 min at room temperature in 25 μL of reaction solution containing: TdT reaction buffer (Roche Applied Science), 2.0 mM CoCl_2_, 10 μM dATP, 2.5 μM Alexa Fluor 488-dUTP (Molecular Probes) and 10 units of TdT (Roche Applied Science). The reaction was stopped with three washes in 2xSSC for 5 min. Slides were mounted using the anti-fading reagent ProLong Gold containing 5 μg/mL DAPI (4′,6-diamidino-2-phenylindole, MolecularProbes). Slides were examined on an Axiobserver microscope (Carl Zeiss), and images were collected at 100× (oil immersion 1.4 NA). Analyses were based on counts of 1000 cells of WT and KAP4 mutants considering the kDNA replication as described by Liu and Englund^55^.

### Electron microscopy

#### Scanning electron microscopy (SEM)

Sample processing was performed using glass coverslips precoated with 1 mg/mL poly-l-lysine. Protozoa were fixed for 1 h in 2.5% glutaraldehyde diluted in 0.1 M cacodylate buffer pH 7.2. Cells were subsequently adhered to coverslips, postfixed for 1 h with 1% osmium tetroxide diluted in cacodylate buffer, and dehydrated in a graded alcohol series (50%, 70%, 90%, and two exchanges of 100% ethanol for 10 min each step). Samples were critical-point dried in a Leica EM CPD030 apparatus (Leica, Wetzlar, Germany). Specimens were sputtered with gold in a Balzers FL9496 unit (Postfach 1000 FL-9496 Balzers Liechtenstein) and observed in an EVO 40 VP SEM (Zeiss, Germany). In all assays performed, approximately 500 cells were observed.

#### Transmission electron microscopy (TEM)

Protozoa were fixed for 1 h in 2.5% type II glutaraldehyde (Sigma, Missouri, USA) diluted in 0.1 M cacodylate buffer, pH 7.2. The protozoa were washed twice in cacodylate buffer and postfixed (1% osmium tetroxide, 0.8% potassium ferrocyanide, 5 mM calcium chloride diluted in 0.1 M cacodylate buffer) for 1 h. Samples were then washed in cacodylate buffer, dehydrated in a graded series of acetone solutions (50%, 70%, 90%, and two exchanges of 100% acetone) for 10 min at each step, and embedded in Polybed resin. Ultrathin sections were stained with 5% uranyl acetate for 45 min and lead citrate for 5 min before observation in a Jeol 1200 EX TEM operating at 80 kV. In all assays performed, approximately 500 cells were analyzed.

### Damage quantification by long-range qPCR analysis

Parasite cultures were treated with the respective drug as reported above. After treatment, 1 × 10^8^ cells were harvested by centrifugation at 3000*×g* for 5 min at the time points after treatment indicated on the graph. The first time point (0 h) was collected immediately after the end of UV radiation exposure, and after the washes to remove cisplatin from the media in cisplatin treatment. DNA extraction was performed by using the QIamp^®^ DNA Mini and Blood Mini Kit (Qiagen, cat: 51104) protocol for tissue extraction.

Amplification was performed using a Kappa LongRange HotStart PCR Kit (Sigma, cat: KK3501). Specific primers for the mitochondrial coding region were used and are listed in Table 1. Amplification of the large mitochondrial fragment (approximately 10 kB) was performed by using primers qPCRMitF and qPCRMitR. Amplification of the small mitochondrial fragment (250 bp) was performed by using the primers qPCRMitSmF and qPCRMitR. For the nuclear fragment analyses, the amplification of the larger fragment was performed using the primers qPCRNucF and qPCRNucR. The smaller fragment was amplified using the primers qPCRNucSmF and qPCRNucR.

The assay consists of the comparison of the amount of amplified material of treated cells with the amount of amplified material within nontreated cells. The smaller fragment was used to normalize the amplification of the large fragments and to avoid any bias from uneven loading of template DNA among the various PCRs. The normalized value of treated and nontreated cells was compared, and the relative amplification was subsequently calculated. These values were used to estimate the average number of lesions/10 kb of the mitochondrial genome using a Poisson distribution. All the results presented are the mean of two technical replicates of amplification and two different biological experiments. Details of the data analysis can be found in the literature^56^.

## Results

To allow genetic manipulation in *A. deanei* facilitated by CRISPR-Cas9, we first generated an *A. deanei* mutant expressing SpCas9 and T7RNAPol by transfecting log-phase cells with the pTB007, generously provided by Dr. Eva Gluenz and previously used to generate a similar mutant in *Leishmania* sp^52^. Western blotting confirmed SpCas9 expression in the mutants, using *L. mexicana* T7RNAPol-SpCas9 as a control (Supplementary Information 1).

**Figure 1:**
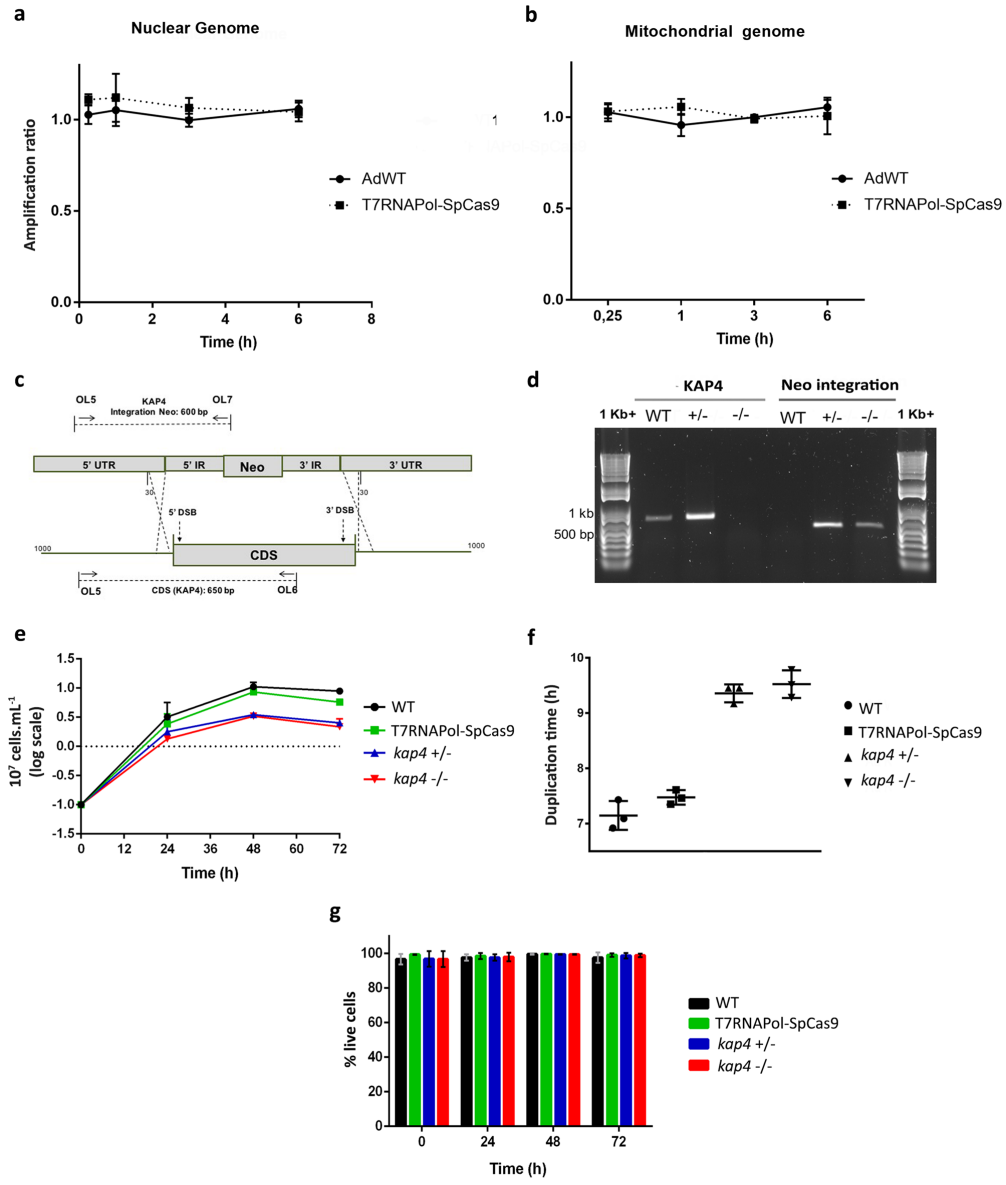
Generation of *KAP4* mutants, cell proliferation and viability. qPCR amplification showing that there was no damage to the nuclear (**a**) and mitochondrial (**b**) DNA of the T7RNAPol-SpCas9 cells compared to WT cells. (**c**) Diagram representing the sgRNA PCR transfection that allows for double strain breaks (DSBs) at the 5′ and 3′ ends of the genes and repair-templates mediated recombination at the UTRs 30 nt upstream and downstream of the CDS. Diagnostic PCR oligonucleotides were designed to amplify the integrated NEO repair template, binding upstream of the open reading frame (OL5) and internally to the NEO gene (OL6), and the presence (WT and +/−) or absence (−/−) of *KAP4* (650 bp, OL5 + OL7). (**d**) Diagnostic PCR showing *KAP4* gene deletion and Neo selectable marker integration in the *A. deanei* genome. (**e**) Growth curve for 72 h showed that *KAP4* mutants present a reduced proliferation in relation to WT and T7RNAPol-SpCas9 strains. Cell number was plotted on a logarithmic scale, and the presented data are the mean ± s.d. of three independent cell cultures. After 48 h, when cells reached the peak of the exponential phase, a paired T test (p < 0.05) was performed to compare control and mutant cells. (**f**) Duplication time of WT, T7RNAPol-SpCas9 and cells deleted for *KAP4*. (**g**) The cell viability was similar among the strains analyzed and maintained even after 72 h of cultivation. The presented data is a mean ± s.d. of three independent cell cultures. WT, wild-type cells, *kap4*^+⁄−^, cells with deletion for one allele, *kap4*^−⁄−^, null mutant.

To verify whether the expression of SpCas9 in the AdT7RNAPol-SpCas9 strain could constitutively cut nonspecific sites, long-range qPCR quantification was performed to determine the amount of possible accumulation of DNA damage in those cells. WT protozoa were used as a controls, since they do not contain the cassette construction for the SpCas9 expression. If SpCas9 generated nonspecific DNA damage, it was expected to produce a difference between the amplification ratio of the genetically modified strain in comparison with WT cells. The amplification for both strains was approxemetly 1, indicating that the expression of SpCas9 on *A. deanei* did not generate DNA strand breaks in a nonspecific manner in either nuclear or mitochondrial genomes (Fig. 1a,b). The confirmed mutant had a regular morphology, and SpCas9 expression was well tolerated. To delete *KAP4*, *A. deanei* was cotransfected with a repair template containing the neomycin resistance gene and 30 nt homologous to flanking *KAP4* UTRs′, and 2 sgRNA templates were expressed in vivo by T7 RNA polymerase to insert DSBs at the 5′ and 3′ ends of the gene. Cells were kept under G418 pressure and mutants were confirmed by diagnostic PCR to detect the resistance cassette integration and *KAP4* deletion (Fig. 1c). We were able to disrupt one or both alleles of *KAP4* by integrating a resistance marker (*NEO*), and enabling selection with neomycin (Fig. 1d), thereby successfully validating our system.

Analyses of cell proliferation showed that WT, T7RNAPol-SpCas9 and *KAP4* mutants cultivated for 48 h, which corresponds to the peak of exponential phase, presented different proliferation profiles: when compared to WT protozoa, T7RNAPol-SpCas9 strain had a reduction of 19% in proliferation, whereas these values were equivalent to 67% and 69% to gene-deficient cells for one or both alleles of *KAP4*, respectively (Fig. 1e). The duplication times of WT and T7RNAPol-SpCas9 were similar and equivalent to 7.1 and 7.4 h, respectively, whereas values obtained for Δ*kap4* with single or double deletions were 9.3 h and 9.5 h, respectively (Fig. 1f). Although WT cells, as well as the T7RNAPol-SpCas9 background and *KAP4* mutants, exhibited distinct decreases in proliferation after 48 h (Fig. 1e), the viability rate after 72 h of cultivation was similar to that of all cell types, that is, approximately around 98.5% (Fig. 1g).

**Figure 2:**
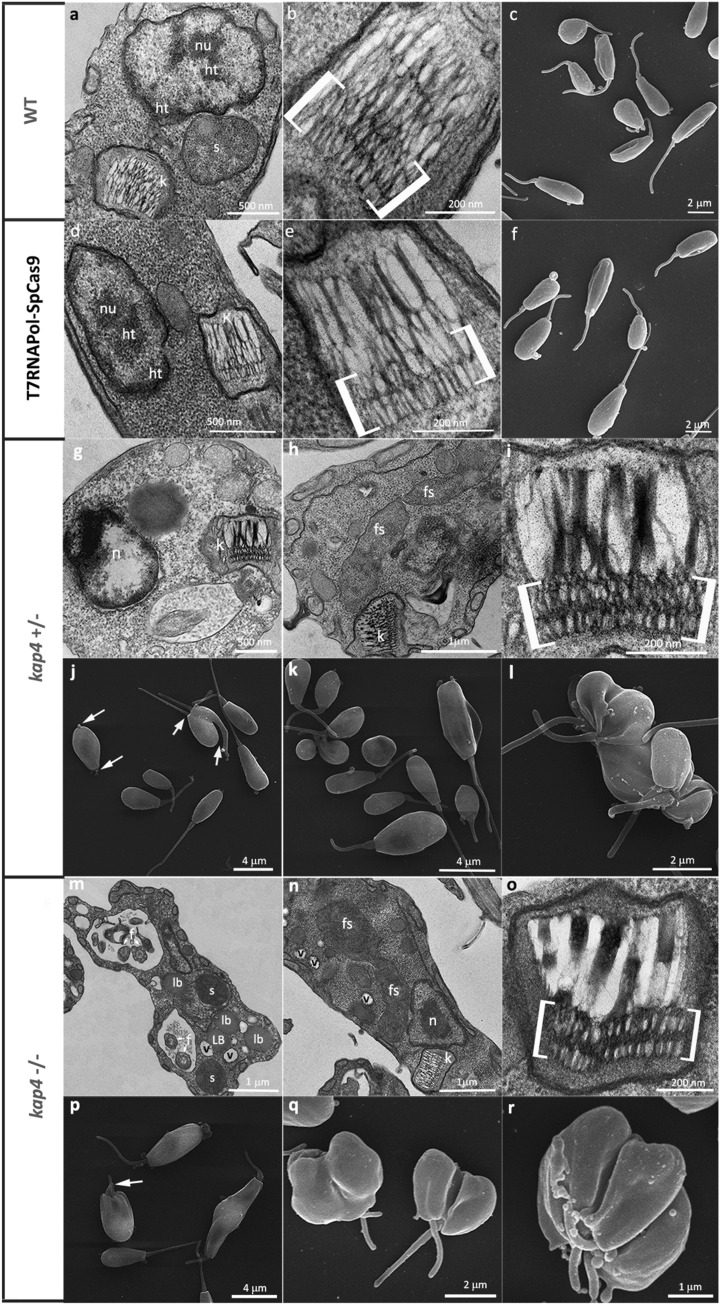
Ultrastructure and morphology of *A. deanei*. WT (**a**–**c**), T7RNAPol-SpCas9 (**d**–**f**) and *KAP4* mutant cells with single (**g**–**l**) or double deletions (**m**–**r**). (**a**,**b**) Transmission electron microscopy of WT cells showed typical characteristics of symbiont-harboring trypanosomatids, which were also observed in T7RNAPol-SpCas9 cells (**d**,**e**). *kap4*^+⁄−^ and *kap4*^−⁄−^ cells presented ultrastructural alterations as a high condensation of nuclear DNA (**g**), a densely packed kDNA (**i**–**o**), a filamentous symbiont (**h**,**n**), dividing cells with two flagella in the same flagellar pocket (**m**). Scanning electron microscopy showed the typical choanomastigote form in WT and T7RNAPol-SpCas9 cells of mutant cells (**c**,**f**). *kap4*^+⁄−^ mutants presented ultrastructure alterations such as asymmetric division (**j**, yellow arrow), which generated cells with different dimensions (**k**) and protozoa with multiple cell bodies and flagella (**l**). *kap4*^−⁄−^ cells presented cytokinesis impairment that generated a popcorn-like phenotype (**q**–**r**). In both mutant strains, cell bodies and flagellum shortening were observed (**j**,**p**, white arrows). *ht* heterochromatin, *k* kinetoplast, *lb* lipid body, *n* nucleus, *nu* nucleolus, *s* symbiont, *f* flagellum, *fs* filamentous symbiont, *v* vacuole. Brackets show the more densely packed kDNA.

The morphological and ultrastructural analyses in this study used cells cultivated for 24 h, which is equivalent to the exponential growth phase of *A. deanei,* whose generation time is equivalent to 6 h. Transmission electron microscopy images showed that as in other trypanosomatids, the nucleus usually occupies a central position in the cell body and contains a nucleolus surrounded by heterochromatin, which is also observed at the nuclear periphery. The symbiont was usually observed close to the host cell nucleus and delimited by two membranes (Fig. 2a). *A. deanei* WT displays a trapezoidal kinetoplast containing a looser arrangement of the kDNA fibers in the central area and a more densely packed array in the region that faces the TAC and connects the mitochondrial DNA to the basal body (Fig. 2b). This same phenotype was observed in the CRISPR-Cas9 background cell line that did not have alterations in kinetoplast shape or kDNA arrangement (Fig. 2d,e). Scanning electron microscopy demonstrated that the WT and CRISPR-Cas9 background strains presented the typical choanomastigotes of the *Angomonas* genus. The smooth cell surface often exhibited gentle undulations that corresponded to mitochondrial branches (Fig. 2c–f).

In cells with a single deletion (*kap4*^+⁄−^) the kinetoplast shape was maintained; however, kDNA fibers of the central area were broken in most cells, and the kinetoplast network was determined to be more condensed as a whole than those observed in control cells. In some instances, the nucleus presented matrix loss and a more condensed chromatin (Fig. 2g,i). Such protozoa showed unusually elongated symbionts, indicating that bacterial division was impaired (Fig. 2h). In *KAP4* null mutants, cells with division impairment phenotype usually presented two flagella in the same flagellar pocket (Fig. 2m). The symbiotic bacterium was also affected in these cells, which presented filamentous forms surrounded by small vacuoles (Fig. 2n). The kDNA packing was severely compromised in the whole network, especially in the central area (Fig. 2o). Alterations in the nuclear ultrastructure were rarely observed.

As a next step, analysis by scanning electron microscopy was performed by comparing *KAP4* mutants and WT protozoa. Cells with a single gene deletion (*kap4*^+⁄−^) had alterations in morphology, with many protozoa showing a round shape with a shortened flagellum (Fig. 2j, white arrow). Part of the culture presented body shape asymmetry during division (Fig. 2j, gray arrow), which resulted in the generation of daughter cells with different dimensions (Fig. 2k). Protozoa with multiple cell bodies and flagella were also observed, indicating cytokinesis impairment (Fig. 2l). Null mutants also presented morphological alterations, such as cell body shortening and flagellar length reduction (Fig. 2p). A high number of cells with impaired cytokinesis was observed, thereby generating a popcorn-like phenotype (Fig. 2q–r).

**Figure 3:**
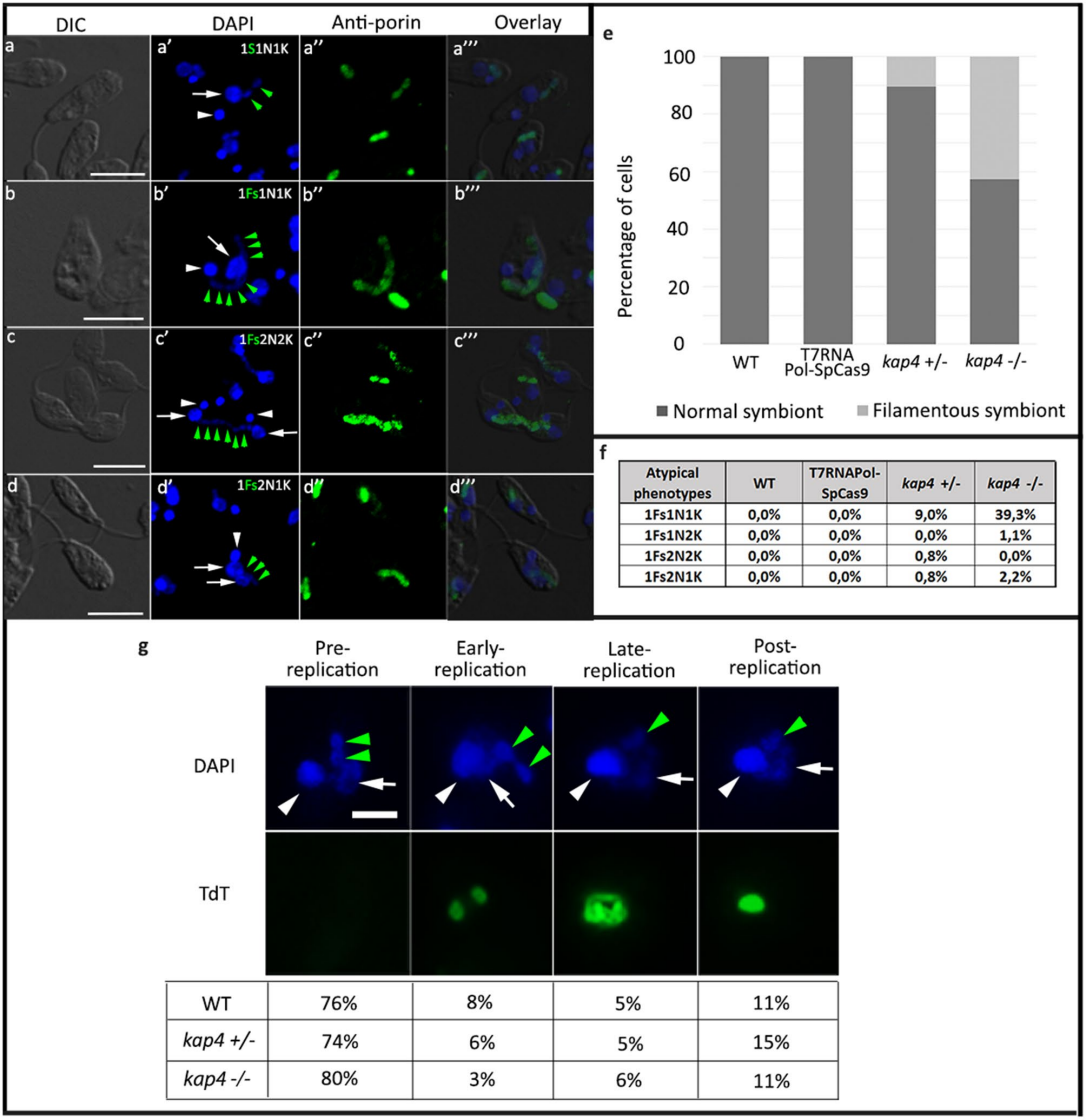
Atypical phenotypes were observed in *KAP4* mutant cells cultivated for 24 h after labeling with DAPI and anti-porin antibodies. WT (**a**–**a″**); *kap4*^+⁄−^ mutants containing one filamentous symbiont with multiple nucleoids (Fs—green arrowhead), one nucleus (N-white arrows) and one kinetoplast (K-white arrowhead) (**b**–**b″**) or two nuclei and two kinetoplasts and (**c**–**c″**); *kap4*^−⁄−^ cells were seen with one filamentous symbiont, two nuclei and one kinetoplast (**d**–**d″**). Bars 5 μm. (**e**) Counting of cellular patterns showing that filamentous symbionts (Fs) are more frequent in *kap4*^−⁄−^. (**f**) Percentage of cells presenting atypical phenotypes. (**g**) In situ labeling showing the different stages of kDNA network replication in WT and mutant cells (according to Liu and Englund 2007)^15^. Green arrowheads indicate the symbiont, white arrows the nucleus and white arrowheads the kinetoplast. Bars 1 μm. t test p-value < 0.005. A total of 1000 WT and KAP4 mutant cells were counted in 3 independent experiments.

Analyses of cellular patterns were performed in *A. deanei* labeled with DAPI and with an anti-porin antibody that recognizes the endosymbiont, considering the number of nuclei, kinetoplasts and symbionts, as well as the shape of the bacterium (Fig. 3). As expected, in asynchronous cultures of WT cells, approximately 30%, presented one rod-shaped symbiont, one kinetoplast and one nucleus (1S1K1N). Most cells, that is, approximately 50%, also presented 1S1K1N; however, the symbiont presented a constricted or dividing format. The other part of the culture, approximately 20%, was composed of cells containing two rod-shaped symbionts. Such protozoa presented one or two kinetoplasts and nuclei; however, kinetoplast division was always observed before the karyokinesis. In *KAP4* mutants cultivated for 24 h, protozoa presented atypical phenotypes as two nuclei, one kinetoplast and one filamentous symbiont (1Sf2N1K) or two nuclei, two kinetoplasts and one filamentous symbiont (1Sf2N2K), an indication of kDNA division and cytokinesis blockage, respectively (Fig. 3a–d). In KAP4 mutants cultivated for 24 h, filamentous symbionts were observed in 3% *kap4*^+*⁄−*^ cells and in 54% of *kap4*^*−⁄−*^ protozoa, exhibiting bacterium division impairment (Fig. 3e).

The counting of cell patterns in KAP4 mutants showed that the percentage of filamentous symbionts was higher in cells containing one bacterium, one nucleus and one kinetoplast (1Sf1N1K) than in cells containing two nuclei or two kinetoplasts, indicating that as the cell cycle progresses, the symbiont filamentation increases, eventually leading to bacterial lysis. The percentage of cells containing one filamentous symbiont, two nuclei and one kinetoplast (1Sf2N1K) was almost three times higher than in *kap4*^*−⁄−*^ protozoa when compared to *kap4*^+*⁄−*^ cells, indicating that in the double mutant, kinetoplast division was more affected (Fig. 3f). To check whether the KAP4 mutant phenotype has an impact on kDNA replication, assays of dUTP incorporation by the deoxynucleotidyl transferase terminal (TdT) were performed. The results showed that the percentage of cells with the kDNA in the early replication stage was 62.5% lower in cells containing deletions of both *KAP4* genes than in WT protozoa. During this stage, the kinetoplast exhibits strong labeling in the antipodal sites but little labeling in the kDNA network (Fig. 3g).

**Figure 4:**
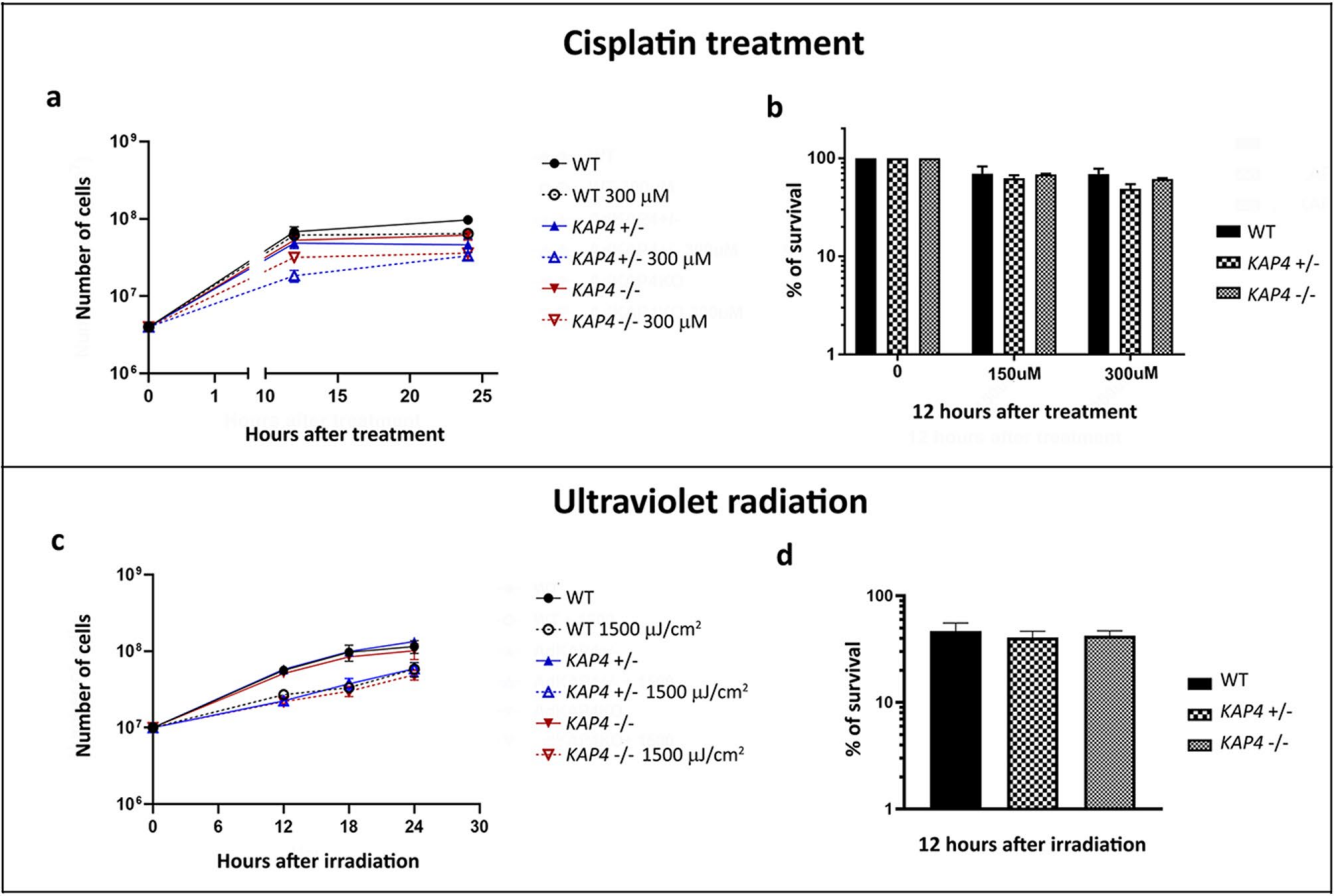
Cell growth and survival after cisplatin treatment or UV radiation. After 12 h, no remarkable differences were observed in cell proliferation and survival when comparing WT and mutant protozoa after treatment with 150 μM and 300 μM cisplatin (**a**,**b**) or exposure to UV radiation (**c**,**d**).

Considering the structural results obtained in this work, we assumed that *A. deanei* KAP4 could participate in kDNA metabolism. To confirm this hypothesis, WT and mutant cells were exposed to cisplatin or UV radiation to verify the cell response to DNA damage. These agents cause distortions in the DNA that can impair transcription and replication, with cisplatin lesions being more effective than UV light. Protozoa that had one or both *KAP4* genes deleted were able to grow after treatment with cisplatin or exposure to UV, although in cisplatin treatment, the mutant cells presented a slight decrease in cell proliferation compared to the WT strain after 12 h of treatment, especially the single gene-deficient mutant treated with the highest inhibitor concentration (Fig. 4a–d).

**Figure 5:**
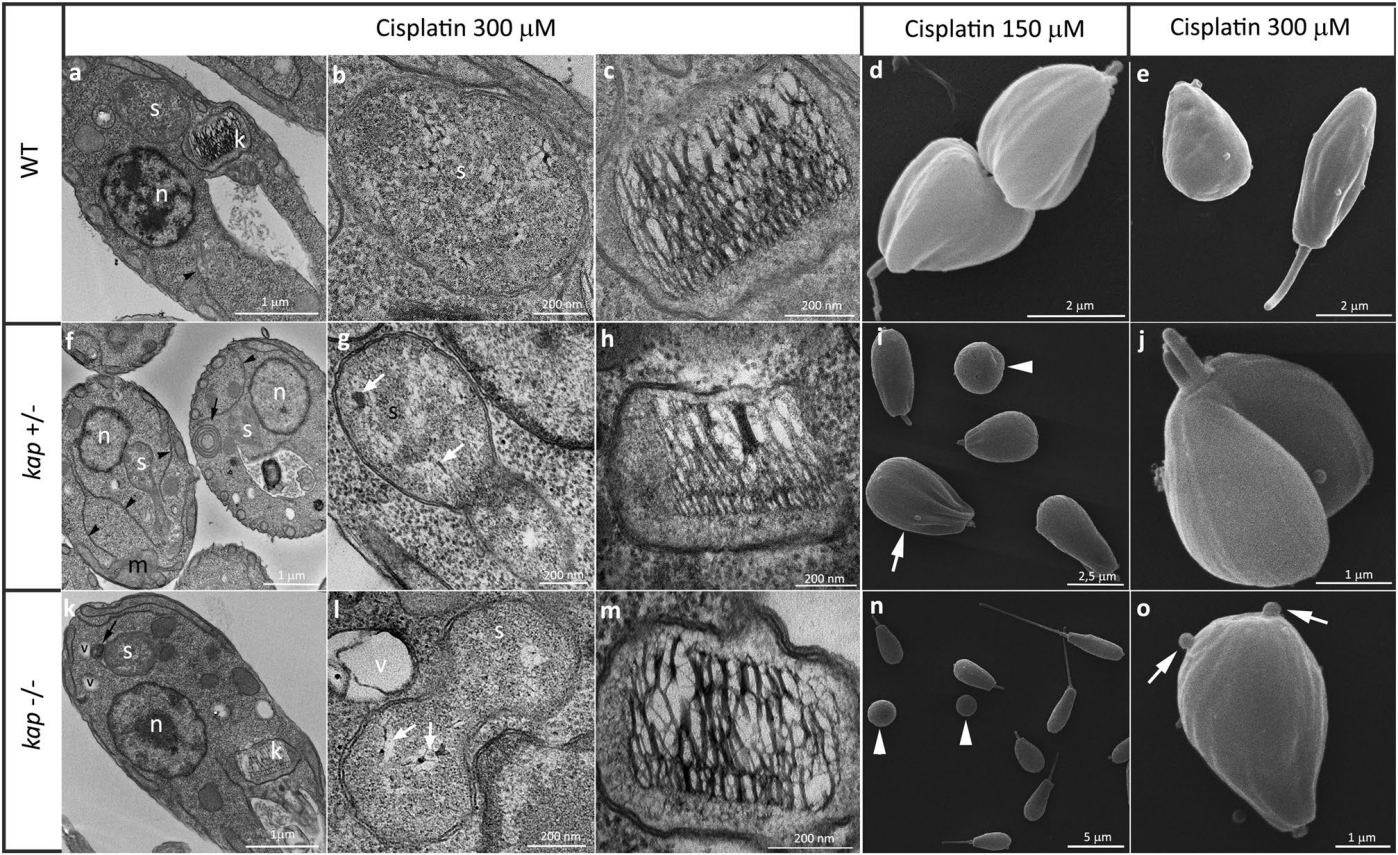
Effects of cisplatin on the ultrastructure of mutant cells as revealed by TEM (**a**–**c**,**f**–**h**,**k**–**m**) and SEM (**d**,**e**,**i**,**j**,**n**,**o**). A–E: WT cells treated with cisplatin did not present ultrastructural alterations by TEM. However, SEM showed rounded cells with a shortening flagellum (**d**,**e**). (**f**–**o**) mutant cells treated with cisplatin. (**f**) Note DNA unpacking in the nucleus (n), the proximity between the ER (black arrowhead) and the endosymbiont, and mitochondrial branch swelling (m). (**g**,**l**) The symbiotic bacterium presents alterations in the nuclear matrix and DNA condensation (white arrows). (**k**,**l**): The symbiont presented membrane convolutions (black arrow) and was surrounded by vacuoles, an indication of autophagy. (**h**,**m**) In mutant cells, the kDNA arrangement was not affected in relation to protozoa not submitted to cisplatin treatment. *n* nucleus, *k* kinetoplast, *m* mitochondrial branch, *s* symbiont, *v* vacuole. (**d**,**e**,**i**,**j**,**n**,**o**) WT and mutant cells of both types treated with cisplatin presented a rounded format containing a shortening flagellum. Other atypical phenotypes, such as fat-cell shape (**d**), lack of flagellum (**i**,**n,** arrowheads) and plasma membrane blebs (**o**, arrows), were also observed.

Considering the cellular morphology and cellular organization, microscopy analyses were performed to test whether *KAP4* mutants presented atypical phenotypes in relation to WT cells after cisplatin treatment. This compound interacts with DNA and proteins and forms intrastrand or interstrand DNA crosslinks that cause distortions in the double helix, thereby blocking duplication and transcription. Transmission electron microscopy images showed that WT cells did not present nuclear or kinetoplast changes after incubation with cisplatin, even when a higher drug concentration (300 µM) was used (Fig. 5a–c). The same phenomenon was observed in the background T7RNAPol-SpCas9 cell line (data not shown). Similarly, *KAP4* mutants did not display topological rearrangement on the kDNA network compared to WT cells treated with cisplatin (Fig. 5h,m). However, other cellular structures suffered alterations in mutant protozoa. In *kap4*^+⁄−^ cells, nuclear DNA unpacking was observed, as well as myelin figures in the cytoplasm (Fig. 5f, black arrow) and mitochondrial swelling. The abundance of the endoplasmic reticulum was noted and also its frequent association with the symbiont, which sometimes was seen surrounded by this organelle, suggested an autophagic process (Fig. 5f, arrowheads). The symbiont also displayed matrix loss and alterations in its DNA condensation (Fig. 5g, white arrows). In null mutants treated with 300 µM cisplatin, the primary ultrastructural alteration was observed in the symbiont that presented membrane convolutions (Fig. 5k, arrow), matrix loss and densely packed DNA fibers (Fig. 5l, white arrows). It is also worthwhile to mention the presence of vacuoles around the symbiont, indicating that the bacterium had lysed (Fig. 5k–l).

Analyses by SEM showed that cultures of *A. deanei* WT cells presented a higher incidence of rounded protozoa with a shortening flagellum after treatment with 150 and 300 µM cisplatin for 24 h (Fig. 5d,e). This phenotype was also observed in mutant cells after treatment with both concentrations (Fig. 5i,j,n–o, white arrowheads). Protozoa presenting a fat cell-like phenotype and lacking the flagellum (Fig. 5i, white arrow) were observed after treatment with 150 µM cisplatin for 24 h. After using 300 µM of this drug, protozoa with the cytokinesis phenotype were observed more frequently (Fig. 5j), indicating division impairment, as well as plasma membrane blebs at the posterior end of the cell body (Fig. 5o). Protozoa that had one allele deleted seemed to have their morphology more affected than null mutant when treated with this genotoxic agent.

**Figure 6:**
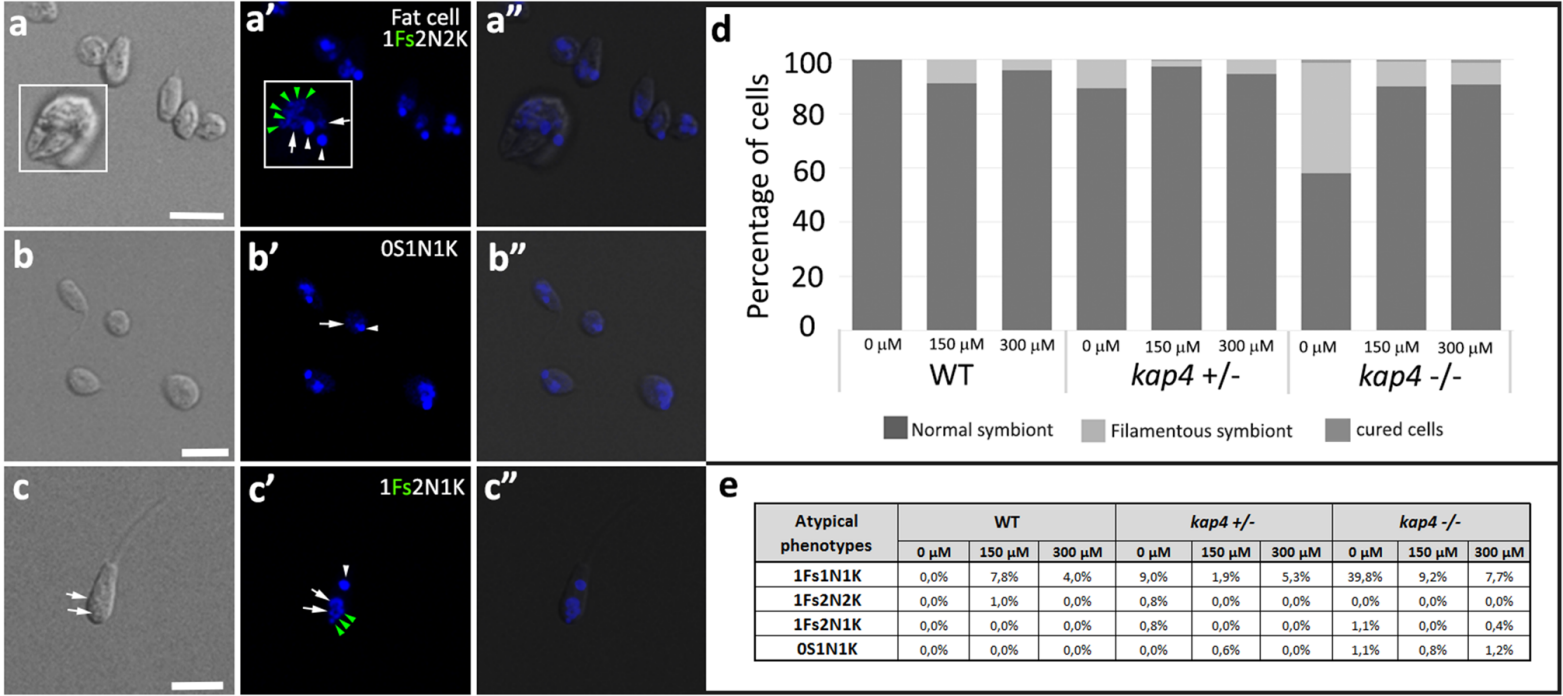
DAPI-stained mutant protozoa presented different atypical phenotypes when compared to WT cells after treatment with cisplatin for over 24 h (**a**–**c**). (**a**–**a**″) WT cells treated with 150 µM cisplatin presented rounded shapes, and the fat cell phenotype contained a symbiont with multiple nucleoids (white square, green arrowheads). (**b**–**b**″) Ad kap4^(+⁄−)^ cells treated with 300 µM cisplatin lacking the symbiont. (**c**–**c″**) Ad kap4^(−⁄−)^ cells treated with 300 µM cisplatin containing one filamentous symbiont, two nuclei and one kinetoplast. (**d**) Counting of cellular patterns considering the presence of normal or filamentous symbionts. (**e**) Percentage of cells with atypical phenotypes. Bars 5 μm. *Fs* Filamentous symbiont., *N* nucleus—white arrows; K-kinetoplast—white arrowheads. A total of 1000 cells of WT and KAP4 mutant cells were counted in 3 independent experiments.

Cells subjected to cisplatin treatment presented atypical phenotypes, as demontrated by fluorescence microscopy analysis. When treated with the lower inhibitor concentration (150 µM), WT trypanosomatids presented rounded shapes with a reduced flagellum length and the fat cell phenotype. Symbionts were seen in the filamentous format, presenting several nucleoids (Fig. 6a–a″). Mutants for *KAP4* treated with cisplatin also presented filamentous bacterium, but in this case, protozoa lacking the bacterium were also observed, as well as cells presenting two nuclei and one kinetoplast (Fig. 6b–b″ and c–c″). Next, we counted the number of protozoa containing a filamentous bacterium after cisplatin treatment for 24 h. In WT *A. deanei,* filamentous bacteria were not identified in non-treated cells, as previously demonstrated. However, after incubation with 150 µM and 300 µM of cisplatin, 14% and 3% of the cells showed filamentous symbionts, respectively. In *kap4*^+/−^ protozoa, when both concentrations of cisplatin were used, the percentage of filamentous bacteria was similar, that is, approximately 2%. In the null mutant (*kap4*^−/−^), values were equivalent to 14% and 8%, respectively (Fig. 6d).

These results indicate that treatment with cisplatin induced symbiont filamentation and that a higher concentration of the inhibitor (300 µM) augmented symbiont lysis, as also suggested by transmission electron microscopy data. Counting of cellular patterns demonstrated that after treatment with cisplatin, the highest percentage of bacterial filamentation was present in the double mutant cells containing one nucleus and one kinetoplast (1Fs1N1K). In these cells, the percentage of protozoa with a filamentous symbiont decreased in a concentration-dependent manner. The percentage of protozoa with bacterial filamentation also decreased with the progression of the cell cycle, as in cells containing two nuclei, reinforcing the notion of the bacterial lysis. Taken together, these data indicate that somehow genotoxic agents alter the cell division pattern in *A. deanei* and that this effect is exacerbated in mutant cells (Fig. 6e). Cisplatin can block replication and trigger checkpoints at the end of S phase and the beginning of G2 to repair lesions, thereby causing cell cycle arrest. However, when cells treated with cisplatin were submitted to flow cytometry analysis, they did not show cell cycle alterations in relation to control cells, even after treatment with 300 µM for 24 h (Supplementary Fig. 2).

**Figure 7:**
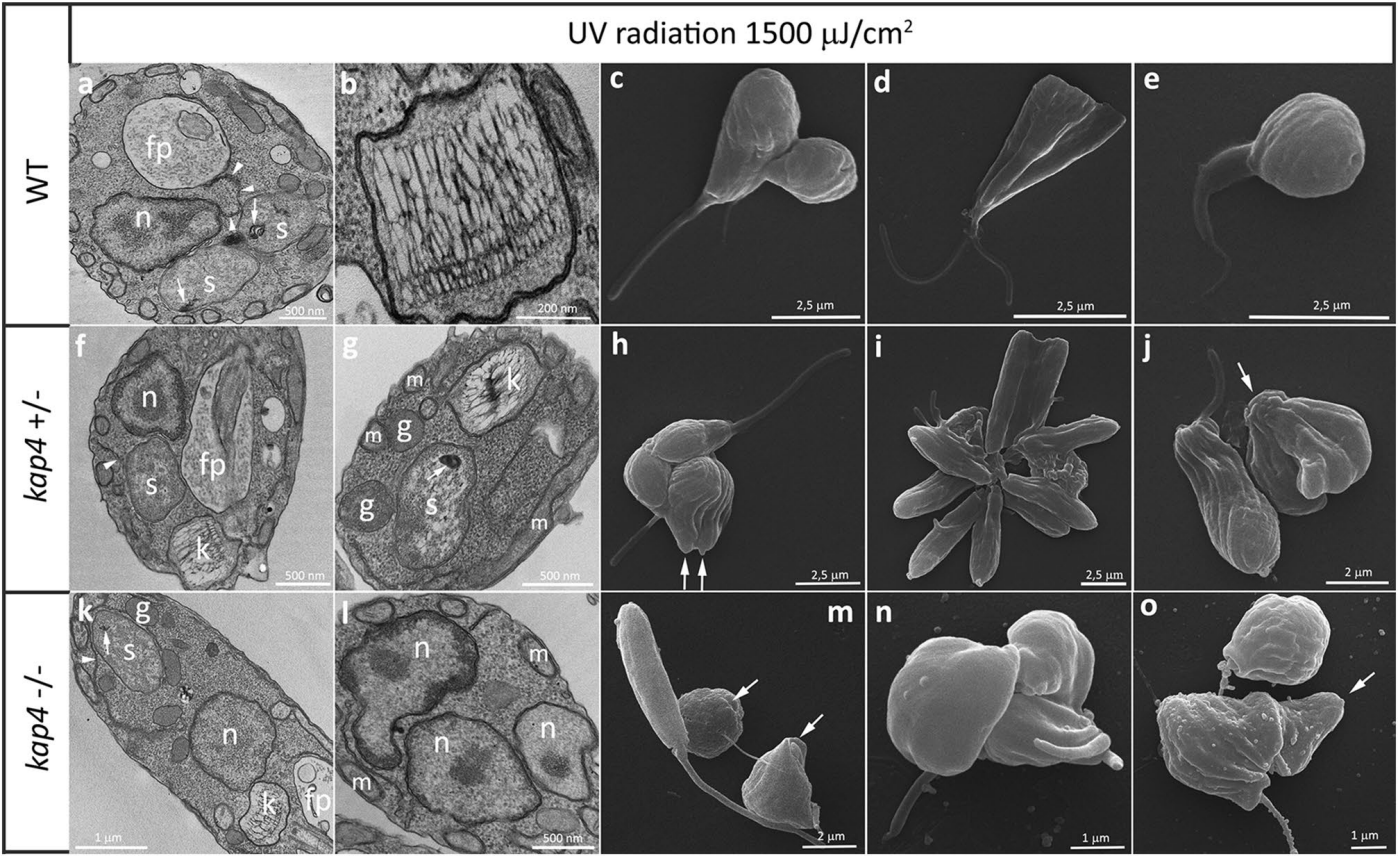
Effects of UV irradiation on the ultrastructure of WT and mutant cells as revealed by TEM (**a**,**b**,**f**,**g**,**k**,**l**) and SEM (**c**–**e**,**h**–**j**,**m**–**o**). (**a**,**b**) WT cells submitted to irradiation. Nuclear DNA condensation and kDNA arrangement were not modified. However, the symbiont genome became densely packed (**a**, arrows). (**f**,**g**,**k**,**l**) mutant cells submitted to UV irradiation. Nuclear DNA condensation and kDNA arrangement were not affected. (**f**,**k**,**g**) The symbiont was seen in association with the ER (white arrowheads), and its DNA suffered condensation (white arrows). (**l**) *kap4*^−⁄−^ cells containing multiple nuclei were also observed. *fp* flagellar pocket, *g* glycosome, *k* kinetoplast, *m* mitochondrion, *n* nucleus, *s* symbiont. (**a**–**c**) WT cells subjected to irradiation presented atypical formats indicating cytokinesis impairment. (**h**–**j**) *kap4*^+⁄−^ cells submitted to UV irradiation presented multiple interconnected cell bodies, indicating that in such mutants, cytokinesis impairment was exacerbated in relation to WT protozoa. (**m**–**o**) *kap4*^−⁄−^ cells submitted to UV irradiation presented a round cell body, and the flagellum was short or even absent (white arrows).

The susceptibility of WT and mutant cells to UV radiation was also verified. Thus, protozoa were subjected to UV-C irradiation, which affects the DNA replication and transcription and can be repaired by nucleotide excision. Ultrastructural and morphological analyses were performed after 24 h of protozoa irradiation at 1500 μJ/m^2^. The results obtained by transmission electron microcopy were similar to those observed for WT and *KAP4* mutant cells treated with cisplatin: nuclear DNA and kDNA did not suffer additional topological alterations in relation to nonirradiated cells (Fig. 7a,b,f,g,k), and a close association of the ER with the symbiont occurred frequently, strongly indicating autophagy (Fig. 7a,f,k, white arrowheads). Notably, after irradiation, mutant cells presented bacteria with a higher DNA condensation (Fig. 7a,g,k, white arrows). Furthermore, polynucleated cells were observed (Fig. 7l).

Scanning electron microscopy analyses showed that WT protozoa suffered morphological alterations after irradiation, exhibiting wrinkled cell surfaces and irregular forms that indicated cytokinesis impairment (Fig. 7c–e). In single- and double-*KAP4*-deleted mutants, the morphological modifications were exacerbated: many protozoa presented multiple interconnected cell bodies, reinforcing the notion that cytokinesis was blocked (Fig. 7h–i). Such cells also presented wrinkled surfaces, and the flagellum was absent in some instances (Fig. 7h,j, white arrows), as also observed in null mutants (Fig. 7m,o, white arrows). In this last case, a high number of round cells were also observed (Fig. 7m–o).

**Figure 8:**
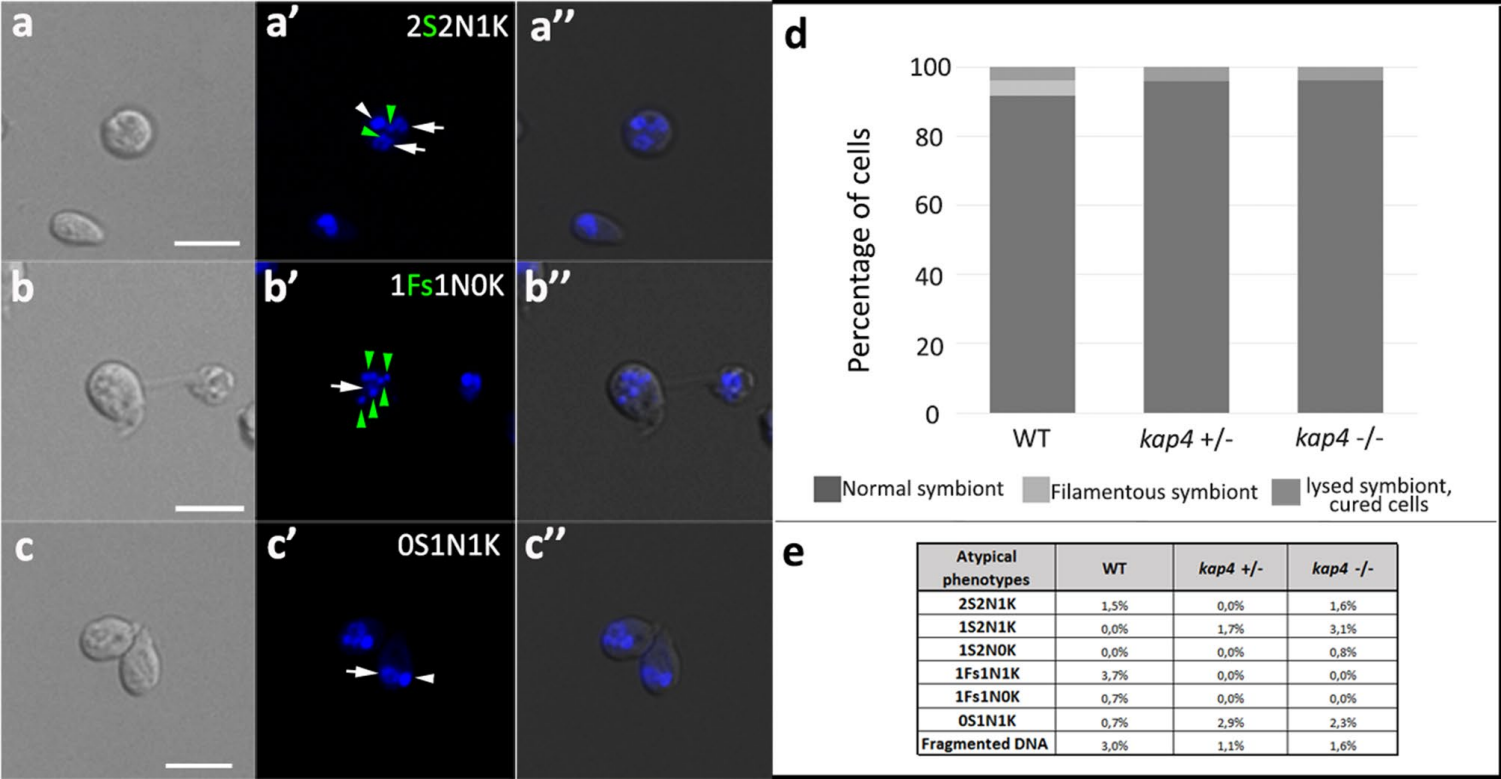
DAPI-stained protozoa exposed to UV irradiation presented atypical phenotypes. Such morphotypes were observed in WT cells (**a**–**a″**), as well as in kap4^+⁄−^ (**b**–**b″**) and kap4^−⁄−^ (**c**–**c″**) mutant cells. (**a**–**a″**) Protozoa harboring two symbionts, two nuclei, and one kinetoplast. (**b**–**b″**) A dyskinetoplastic protozoan, with a filamentous symbiont containing multiple nucleoids and one nucleus. (**c**–**c″**) A cured cell containing one nucleus and one kinetoplast. (**d**) Counting of cellular patterns considering the presence of normal or filamentous symbionts, as well as cured cells (that lost the symbiont) or lysed symbionts. (**e**) Percentage of cells presenting atypical phenotypes. *S* Symbiont and *Fs* Filamentous symbiont, green arrowheads indicate bacterium nucleoids; *N* nucleus—white arrows; *K* kinetoplast—white arrowhead. Bars 5 μm. A total of 1000 WT and KAP4 mutant cells were counted in 3 independent experiments.

Irradiated protozoa were also labeled with DAPI, exhibiting atypical phenotypes that were compatible with asymmetric division and cytokinesis impairment, such as the presence of one kinetoplast and two nuclei in cells containing two symbionts (Fig. 8a) and dyskinetoplastic cells. Such morphotypes were observed in WT cells, as well as in mutant cells (Fig. 8b). Protozoa with filamentous bacterium were observed more frequently in WT cells than in *KAP4* mutants, on which symbiont division was probably more strongly affected. The absence of the symbiont was observed in null mutants (Fig. 8c), which may have been related to the possible occurrence of autophagy, in this case, a symbiophagy, that generated aposymbiotic cells, as suggested by transmission electron microscopy. The very reduced number of WT cells presenting filamentous symbionts (1.4%) and the absence of this phenotype in mutant cells reinforced this notion (Fig. 8d). The percentage of irradiated protozoa presenting atypical phenotypes was low in all cell types (Fig. 8e).

**Figure 9:**
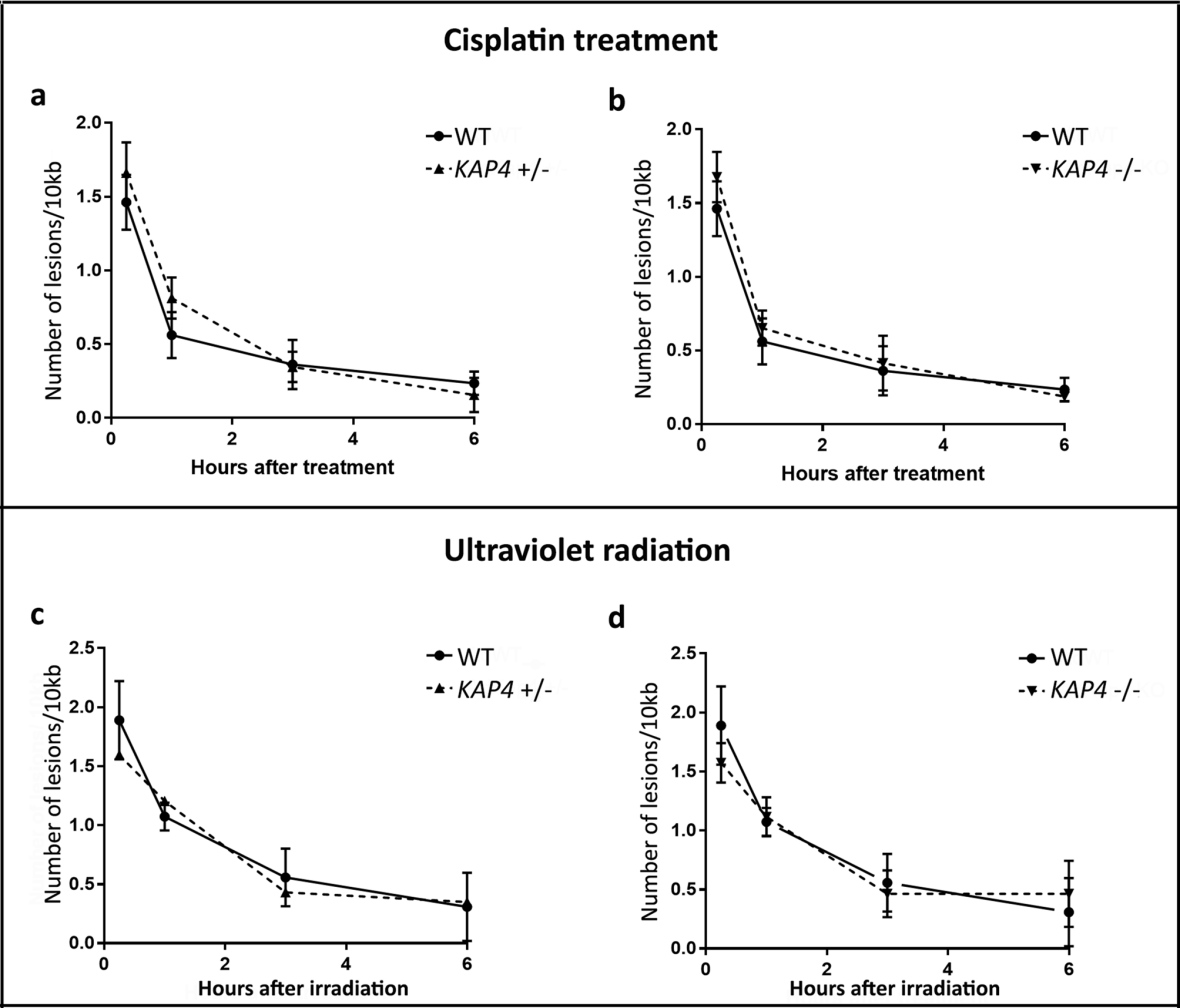
DNA repair kinetics of WT and mutant cells. (**a**,**b**) DNA repair kinetics of WT cells compared to *kap4*^+/−^ cells (left panel) and *kap4*^−/−^ (right panel) after treatment with 300 µM cisplatin. (**c**,**d**) DNA repair kinetics of WT cells in relation to *kap4*^+/−^ cells (left panel) and *kap4*^−/−^ (right panel) after UV damage radiation. As observed for cisplatin, no significant difference was observed in the DNA repair kinetics of all cell types.

To verify whether *KAP4* was involved in kDNA repair, mutant and wild-type cells of *A. deanei* cells were treated with cisplatin and UV radiation, as described before, and DNA repair kinetics were measured by long-range qPCR assay. After treatment with 300 µM cisplatin, WT and both mutant strains presented the same levels of DNA damage on the kDNA, which was approximately 1.5 lesions/10 kB. The repair kinetics were very similar for all cell types: after 3 h of treatment, levels of kDNA damage were almost undetectable, reaching the slowest point after 6 h (Fig. 9a,b, Supplementary Information 3). A similar phenotype was observed for the UV radiation. The levels and DNA repair kinetics of mutant cells were very similar to those observed in WT cells. After 1 h of treatment, most damage had already been repaired, although it required 3 h after UV radiation to reach the same level of repair that was observed in cisplatin-treated cells, with the lowest point being observed at 6 h (Fig. 9c,d). Taken together, these results demonstrate that *KAP4* was not directly involved in the removal of DNA damage generated by cisplatin and UV radiation but, notably, show that lesions generated by both genotoxic agents could be repaired in mitochondrial DNA (Fig. 9a–d).

## Discussion

In recent decades, *A. deanei* has been used as a model to investigate endosymbiosis and the origin of organelles. Genome sequencing is available^26,27,57^, and molecular tools for gene functional studies were developed, although with limited use on the evaluation of gene essentiality and symbiosis maintenance^24,58,59^. The recent application of highly efficient CRISPR-Cas9 protocols to other trypanosomatids, such as *Leishmania* and *T. cruzi* , accelerated functional studies with gene deletion^51,59,60^. In this study, for the first time, we describe gene depletion in an endosymbiont-harboring trypanosomatid. Phylogenetic proximity with *Leishmania* enabled the successful application of the CRISPR-Cas9 system developed by Beneke et al.^51^ to *A. deanei*, resulting in efficient deletion of *KAP4*, a kinetoplast associated protein present in all trypanosomatids so far analyzed^9^.

KAPs can neutralize the negative DNA charge, thus facilitating the interaction of mitochondrial proteins with kDNA, as those involved in replication and transcription. In this work, deletion of *A. deanei KAP4* generated trypanosomatids with reduced cell proliferation and generated cells with atypical phenotypes, as those presenting two nuclei and one kinetoplast, as well as cytokinesis impairment. Cells containing aberrant numbers of nucleus and kinetoplast were also observed in null mutants of *C. fasciculata* for *KAP2* and *KAP3* that presented cell division block^16^. In *T. brucei*, the RNAi knockdown of a kDNA associated protein, resulted in reduced growth and in the appearance of dyskinetoplastic cells^20^. These results reinforce the importance of KAPs to cell proliferation and kDNA network replication in order to guarantee that each new protozoa will receive one kinetoplast during trypanosomatid division.

The coordinated division of the symbiont with the host cell nucleus was previously demonstrated in *A. deanei* and in *Strigomonas culicis*, another symbiont-harboring trypanosomatid^22–24^. In the present work, it was interesting to observe in *KAP4* mutant cells that the kDNA condensation, which is associated with kinetoplast replication impediment, resulted in symbiont filamentation. This filamentation occurred most frequently in mutant with two nuclei and one kinetoplast. Consistent with this notion, TdT labeling showed a lower percentage of *kap4*^*−⁄−*^ cells in the early replication phase when compared to the WT protozoa, indicating that in such cells the mitochondrial DNA replication was delayed or even impaired. Since kDNA loss resulting in dyskinetoplastic protozoa was not observed, it can be assumed that the impediment of mitochondrion DNA replication promoted cytokinesis blockage. Taken together, the results indicate that bacterial division is also coordinated with kinetoplast replication, but further studies are essential to confirm this hypothesis. Cell cycle checkpoints are not well established for most trypanosomatids species, nor are the factors that coordinate the equal partitioning of single copy organelles to daughter cells. Such questions are best studied in *T. brucei*, especially by investigating the role of protein kinases in cell cycle progression, organelle positioning and protozoan morphology^60–64^. Recently, it was shown that *T. brucei* UMSBP2, which is involved in kDNA replication and segregation, is also localized at telomeres. The RNAi system showed that this protein not only participates in nuclear division but also plays a role in the coordinated replication of DNA-containing organelles^65^.

In *A. deanei KAP4* mutants, the high level of kDNA packing was associated with delay in cell proliferation and in kDNA duplication at the early stage, when the covalently closed minicircles are released from the network to initiate replication into the KFZ and then migrate to antipodal sites, where this process continues^4^. Previously, it was shown that the downmodulation of *T. brucei* P93, a kDNA-associated protein localized in antipodal sites, resulted in loss of gapped minicircles and consequently in the network reduction^66^. Similarly, in TbKAP6 RNAi cells, the levels of total minicircles and maxicircles decreased the total amount of nicked/gapped minicircles. In such cells, the kinetoplast presented network shrinkage or elongation, but in both cases, two basal bodies could be identified, indicating failures in kDNA replication and scission. Conversely, in protozoa overexpressing TbKAP6, the minicircle decatenation was enhanced, indicating that a controlled expression of this protein is required for proper kDNA replication^20^.

The kDNA arrangement and metabolism are the result of the coordinated activity of a set of mitochondrial proteins that serve different functions. In addition to KAPs, other proteins are involved in the kDNA replication, such as the minicircle replication factor (MiRF172), which is supposedly involved in the reattachment of replicated minicircles to the kDNA disc. Once depleted, *T. brucei* cells presented reduced kDNA content or even a dyskinetoplastic phenotype^67^. Downregulation of mitochondrial heat shock proteins 70 and 40 also showed impairment of minicircle replication and loss of kDNA, demonstrating the importance of chaperones to the maintenance of the kinetoplast as a cellular structure^68^. In the present work, the generation of dyskinetoplastic cells was not observed among KAP4 mutants. Although the gene deletion promoted increased kDNA compaction, the data obtained by qPCR did not indicate loss of mitochondrial DNA. In UV-irradiated protozoa, a very low percentage of cells without a kinetoplasts was observed.

The DNA repair kinetics showed no differences between *KAP4* mutant cells and the WT strain. In both cases, protozoa were able to efficiently repair the damage generated by cisplatin and UV radiation. In addition, differences in the long-term survival of these cells were not observed. For both genotoxic agent treatments, the kDNA accumulated the same amounts of lesions in WT or *KAP4* mutant cells, suggesting that the topological alterations observed in the kinetoplast network did not affect the susceptibility to DNA damage. It is well established that damage generated by UV radiation and cisplatin is not repaired in humans and other mammalian cells^40,41^. Notably, in *A. deanei*, damage caused by both genotoxic agents on the kDNA was repaired, representing the first demonstration of this type of repair in mitochondrial DNA. The repair kinetics observed in this instance are not related to the kDNA loss, since the number of dyskinetoplastic cells after genotoxic treatment is negligible.

Notably, the DNA repair kinetics were very similar when cisplatin was tested in WT and mutant cells, and the same phenomenon was observed for UV radiation. However, accentuated differences were observed when comparing the two treatments: DNA repair by cisplatin was very fast; thus, after 1 h, most lesions were already repaired, whereas for UV radiation damage, the kinetic is slower. Although it is described that lesions caused by UV and cisplatin are mainly repaired by the nucleotide excision repair pathway, the kinetics observed in this work strongly suggest that lesions caused by each genotoxic treatment activated different and specific responses in *A. deanei*. It is well-known that lesions that block transcription are repaired very quickly compared to other types of lesions. It has also been also reported that the main lesion caused by UV light (thymine dimers) can be tolerated by RNA polymerase^69,70^. In trypanosomatids that present a single mitochondrion, it is possible that a DNA repair pathway associated with transcription exists. The repair of UV lesions may also be associated with the recombination process. In *T. brucei*, it was seen that cells deficient in the Rad51 gene are not able to adequately repair lesions caused by methyl methanesulfonate (MMS)^49,71,72^.

Our structural analyses using microscopy techniques showed distinct atypical phenotypes after treatment with cisplatin or ultraviolet radiation. This phenomenon may be observed because cisplatin can cause more toxic injuries that culminate in cell death. Accordingly, mutant protozoa have higher sensitivity to elevated concentrations of cisplatin and lower percentages of cells containing duplicated nuclei and kinetoplasts than WT or UV-irradiated cells. In mutant cells, this inhibitor promoted a decrease in proliferation and in the number of filamentous symbionts, indicating bacterium lysis. Notably, the cell morphology and growth of KAP4 single allele deletion mutants were more affected by high doses of cisplatin than those of the null mutant cells. Since KAP4 is not an essential protein, it cannot be ruled out that an adaptation process has occurred in cells where both copies of the genes were deleted. A similar phenomenon was observed in null mutants of *Trypanosoma cruzi* and *Trypanosoma brucei* for the MSH2 gene, which encodes a central component of the eukaryotic DNA mismatch repair (MMR) pathway^73^.

In this work, we demonstrated for the first time that the CRISPR-Cas9 system can be used with success to delete genes in *A. deanei*. KAP4 is not an essential protein, but it is involved in the kDNA compaction, leading to the appearance of cells with atypical phenotypes, such as symbiont filamentation and the appearance of two nuclei and one kinetoplast. This protein does not seem to participate in the mitochondrial DNA repair process; however, lesions caused by cisplatin and UV radiation are repaired in the kDNA of this protozoan. The repair kinetics are different for each genotoxic agent, indicating that different pathways are used to repair the lesions. In the case of cisplatin, repair may be associated with transcription.

## Supplementary Information

Supplementary Information.
