# Supplementary material for: Importance of Angomonas deanei KAP4 for kDNA arrangement, cell division and maintenance of the host-bacterium relationship
Source: Sci Rep. 2021 Apr 28;11:9210. doi: 10.1038/s41598-021-88685-8 (PMC8080567; doi:10.1038/s41598-021-88685-8)
Supplement: Supplementary file 1 — Supplementary Information. [file 41598_2021_88685_MOESM1_ESM.pdf]

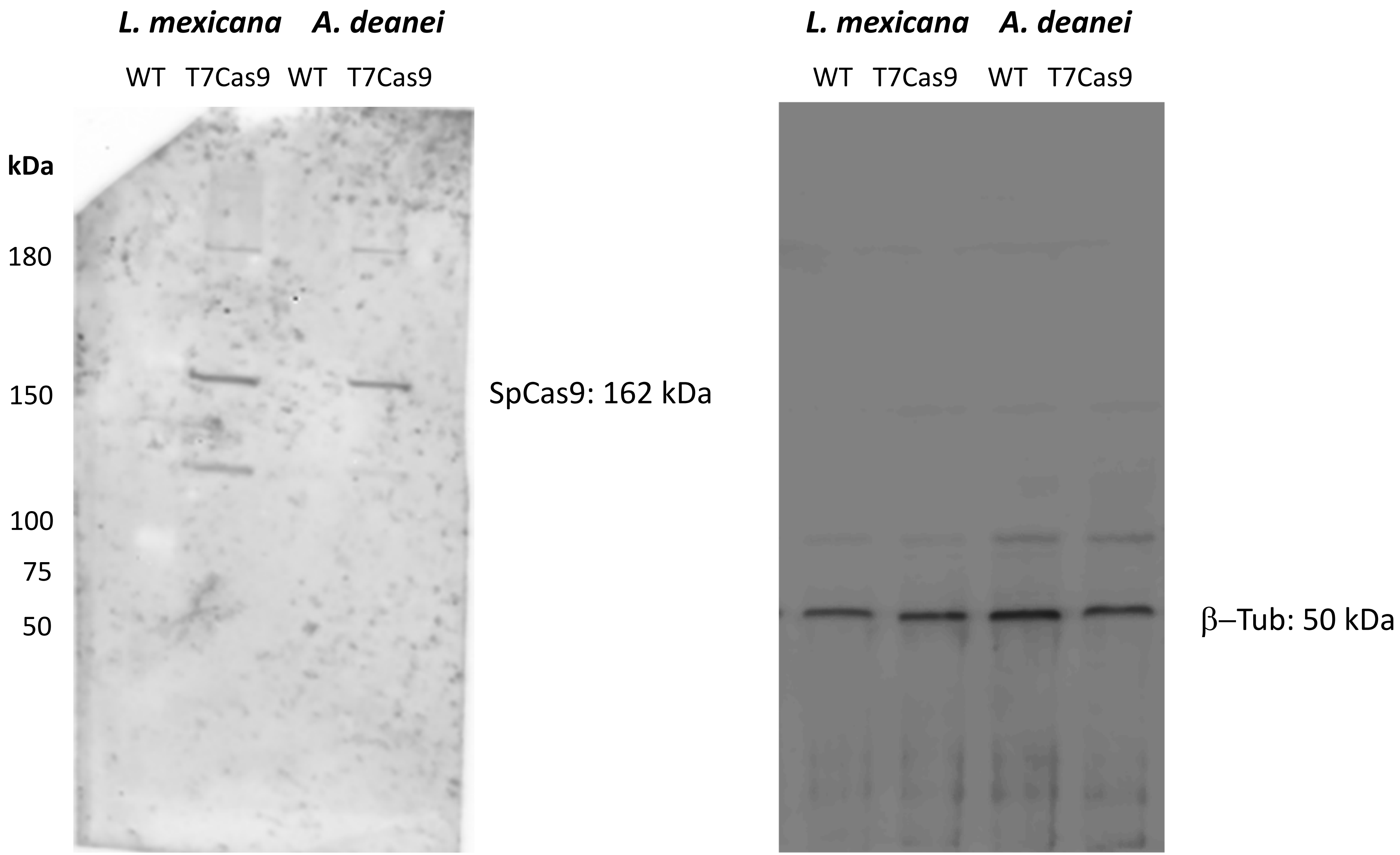

Supplementary Material 1: Total protein extract of WT and T7RNAPol-SpCas9 mutant strains of *A. deanei* and *L. mexicana*, here used as a control, were probed with anti-FLAG antibody (Anti-FLAG M2, Sigma F3165; dilution 1:20,000) for detection of SpCas9 or anti-β-tubulin (Anti-β-Tubulin clone AA2, Sigma T8328; dilution 1:10,000) used as loading control.

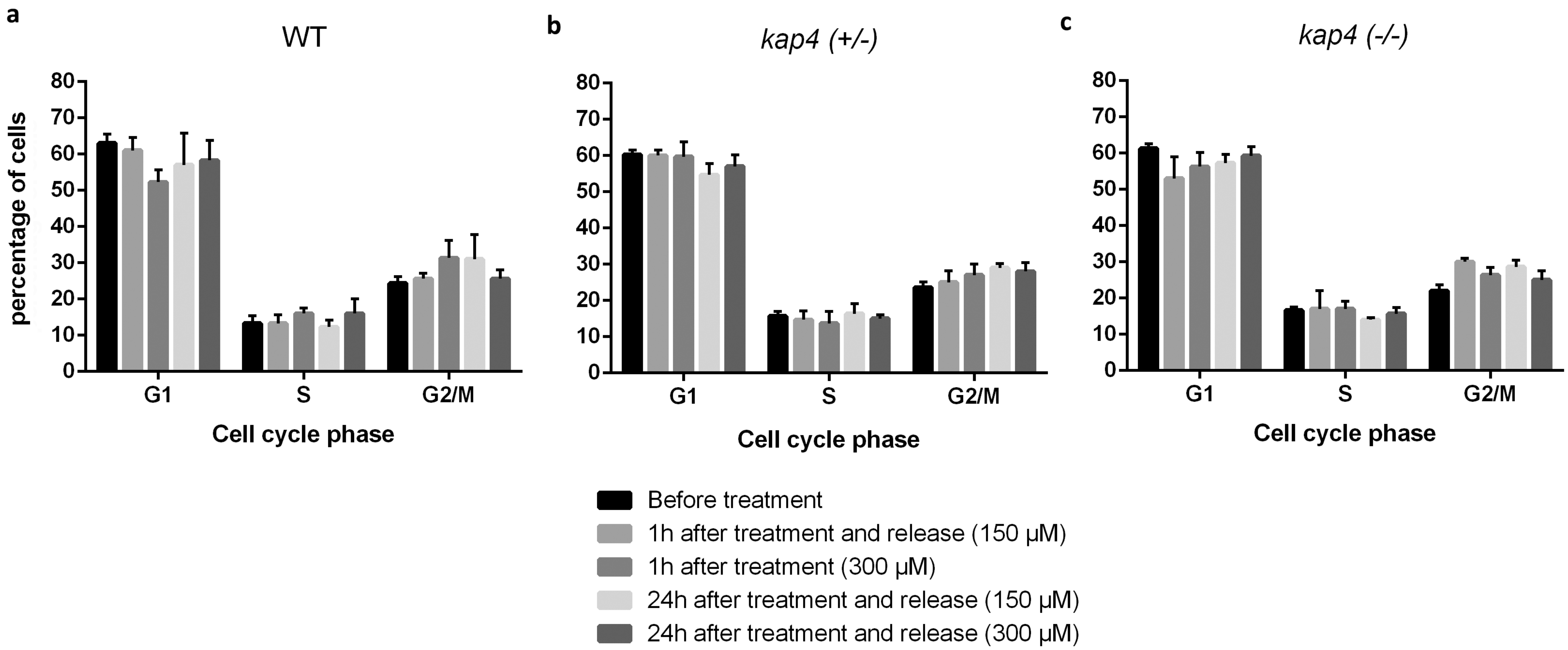

Supplementary Material 2: Flow cytometry analysis of *A. deanei* DNA content in wild type (WT) and mutant cells treated or not with cisplatin for 1 h or 24 h.

Wild type	Replicate 1		Replicate 2	
**Small blank**	1456	1121	1205	1190
**Small NT**	29656	28775	29332	29011
**Small 0,25h**	28744	28312	31223	30774
**Small 1h**	28552	28331	30999	31421
**Small 3h**	27561	28078	30657	30212
**Small 6h**	27771	26987	29044	27908
**Kap4+/-**	**Replicate 1**		**Replicate 2**	
**Small blank**	1987	1999	1024	1016
**Small NT**	30999	31647	32886	32543
**Small 0,25h**	29767	29342	31888	31369
**Small 1h**	30145	29987	29042	29466
**Small 3h**	29688	28921	33112	33876
**Small 6h**	27556	27899	29234	30450
**Kap4-/-**	**Replicate 1**		**Replicate 2**	
**Small blank**	1113	997	1002	996
**Small NT**	26684	27499	29500	29122
**Small 0,25h**	27312	27484	28666	27521
**Small 1h**	25433	26421	29311	29875
**Small 3h**	26911	26041	26884	28333
**Small 6**	25987	26020	26003	27421

Supplementary Material 3: DNA repair kinetics (0.25-6 h) measured by long-range qPCR. Absorbance values for the smaller fragments of A. deanei WT, kap4+/- and kap4-/-.
